# Fabrication of Copper Nanowires Highly Conductive and Flexible Circuits by Direct Ink Writing

**DOI:** 10.3390/ma19030618

**Published:** 2026-02-05

**Authors:** Hui Guo, Haoting Huang, Shijian Shi, Qinghua Sun, Jinping Sun, Kang Liu, Qiang Zhu, Peng Zhang

**Affiliations:** 1 School of Materials Science and Engineering, Harbin Institute of Technology at Weihai, Weihai 264209, China; 2 Comprehensive Research Center of Electronic Information Technology in the MIIT, Weihai 264209, China; 3 Harbin Institute of Technology (Weihai) Qingdao Research Institute, Qingdao 266109, China

**Keywords:** conductive ink, copper nanowires, direct ink writing, flexible circuits, highly conductive

## Abstract

Direct ink writing (DIW) has emerged as a promising method for fabricating flexible electronics. Copper nanowires are a key material for the conductive inks required for this technology. However, copper nanowires suffer from significant challenges, including low aspect ratios, poor oxidation resistance, and difficulty in printing. In this study, a liquid-phase reduction method was used to synthesize copper nanowires with a high aspect ratio (up to 2884) and excellent oxidation resistance. The conductive ink was prepared using ethylene glycol, isopropanolamine (MIPA), and ethanol as solvents. Rheological dynamics simulations were used to investigate the influence of printing parameters on ink printing accuracy, ultimately achieving precise control of the printing process. High-precision copper nanowire flexible circuits with a low resistivity of 2.11 μΩ·cm were fabricated under thermal sintering conditions using the DIW method. These circuits exhibited excellent adhesion, flexural behavior, and water resistance, demonstrating significant practical significance for the low-cost fabrication of high-precision flexible electronic devices.

## 1. Introduction

Flexible electronic printing technology, an innovative manufacturing method in microelectronics, has attracted significant attention in fields such as flexible electrodes, solar cells, antennas, displays, and sensors [1,2,3]. At present, inkjet printing has become a traditional technology for fabricating flexible electronic devices [4,5]. However, the complex and expensive processes have greatly limited its practical application [6]. Compared with traditional technologies like inkjet printing, direct ink writing (DIW) is significantly more convenient to operate and does not require expensive tools. Therefore, it has emerged as a promising alternative [7,8].

Conductive inks are crucial materials for printed electronic devices, combining fluidic properties with electrical conductivity to form conductive layers on various substrates via printing techniques. Although a variety of conductive inks have been developed for flexible printing, existing inks are still limited by relatively low electrical conductivity and poor environmental stability. Gold and silver materials exhibit high electrical conductivity, but their high cost hinders widespread application. Compared to these metals, copper’s bulk price is significantly lower than that of gold and silver. As a raw material for synthetic conductive fillers, copper holds greater potential for low-cost and large-scale mass production. However, sintering shrinkage reduces the interparticle connections, thus affecting conductivity [9,10,11]. Copper nanowires combine low cost with excellent conductivity, thus emerging as an ideal candidate material for conductive inks [12,13,14]. Nevertheless, copper nanowires are prone to oxidation in air, which significantly reduces the conductivity of printed circuits [15,16,17]. In addition, copper nanowires with high aspect ratios tend to aggregate and sediment, affecting the printing stability of conductive inks. Despite significant progress in the development of copper nanowire inks in recent years, further exploration is necessary to achieve conductive inks with high printing stability and conductivity that are suitable for direct ink writing.

The rheological properties of conductive inks and printing process parameters are crucial for high-precision printing of circuits. Screening the optimal ink formulations and process parameters through experiments is time-consuming and costly, while finite element simulation technology is an effective method for optimizing printing performance [18,19,20]. It relies on computer modeling and numerical calculation to simulate the flow behavior of inks during the printing process [21,22,23,24]. Agassant et al. used a Newtonian fluid model to analyze the geometric morphology of a single filament during printing [25]. Some studies have adopted the Carreau model or non-Newtonian power-law fluid model for computational simulation [26,27], but these models cannot reflect the characteristics of yield stress fluids. The Herschel–Bulkley model is another viscoplastic non-Newtonian model that combines the Bingham model with power-law fluids, enabling more accurate fitting of the rheological properties of printing inks. Huang et al. used the Herschel-Bulkley model to study the influence of shear-thinning properties on the flow distance, free surface profile, and final sediment of mud flows [28]. In our previous work, the Herschel–Bulkley model was employed to investigate the effect of direct ink writing process parameters on the printing precision of hydrogel scaffolds, which could accurately reflect the rheological behavior of hydrogels and optimize the printing process parameters. Despite these studies, few researchers have focused on the extrusion printing behavior of conductive inks. Furthermore, the computational fluid dynamics (CFD) model has never been used to simulate the circuit morphology formed by the extrusion printing of conductive inks; it is a way to use a computer to simulate how a fluid moves.

In this paper, copper nanowires with a high aspect ratio and oxidation resistance were synthesized by a liquid-phase reduction method. The liquid-phase reduction method can promote the oriented growth of crystals along specific crystal planes, thus synthesizing copper nanowires with a high aspect ratio. The moderate reducing capacity of ascorbic acid enables the stepwise regulation of reaction kinetics, which is beneficial for controlling the uniform morphology of copper nanowires [29,30,31]. Oleylamine was employed not only as a structure-directing agent to guide the growth of copper nanowires but also to form an oxidation-resistant protective layer on the surface of copper nanowires, achieving high aspect ratios and oxidation resistance of copper nanowires. Meanwhile, a polyol solution system was introduced to prepare copper nanowire conductive inks. Ethylene glycol, with its good polarity, can reduce the agglomeration of copper nanowires. Moreover, the hydroxyl groups of isopropanolamine can form hydrogen bonds with ethylene glycol [32], further inhibiting the agglomeration of copper nanowires, thus enabling the synthesis of stable conductive inks. Isopropanolamine can reduce part of the copper oxides to metallic copper during the sintering process, improving the oxidation resistance of the ink [33]. In addition, a synergistic approach combining experimental analysis and CFD simulation was used to investigate the extrusion dynamics of the ink and the morphology of the circuits. This strategy effectively addresses the key issues in high-precision and high-performance printing of copper nanowire circuits. The developed copper nanowire circuits exhibit excellent electrical conductivity and operational stability.

This study aimed to fabricate copper nanowire flexible circuits with high conductivity and high printing precision. Copper nanowires were synthesized using a liquid-phase reduction method, and a highly stable copper nanowire ink was prepared using a solvent system consisting of isopropanolamine, ethylene glycol, and ethanol. The copper nanowire circuit-forming experiments were conducted by DIW technology, combined with fluid dynamics simulation technology to achieve precise control over the printing process. Furthermore, flexible circuits with low resistivity were fabricated under hot-sintering conditions. Finally, the adhesion, bending deformation tolerance, and cyclic mechanical stability of the circuits were evaluated. The results of this study are expected to enhance the applicability of high-precision flexible electronic devices and are of great significance for promoting the development of wearable electronic devices.

## 2. Materials and Methods

### 2.1. Materials

Copper chloride (CuCl_2_), oleylamine (OM), ethanol, isopropanolamine, ascorbic acid (LAA), and ethylene glycol (EG) were all purchased from Shanghai Aladdin Biochemical Technology Co., Ltd (Shanghai, China).

### 2.2. Synthesis of Copper Nanowires

**Figure 1:**
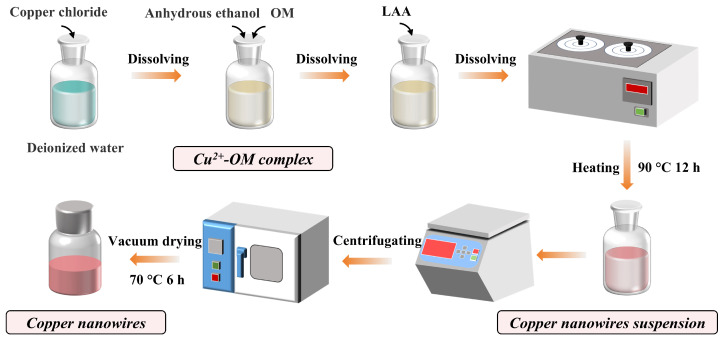
Schematic diagram of copper nanowires fabrication.

Different reaction parameters (copper salt concentration, reducing agent concentration, structure-directing agent concentration) were set to explore the influence of reaction parameters on the morphology and aspect ratios of copper nanowires, as shown in Table 1. Figure 1 shows the process flow of copper nanowire preparation. First, dissolve 0.682 g of CuCl_2_ in 380 mL of deionized water in a glass bottle to obtain a 0.013 mol/L CuCl_2_ solution. Then add 20 mL of anhydrous ethanol, 2.4 mL of OM (C18 80–90%), and 20 mL of a 0.199 mol/L LAA solution. Subsequently, the solution was mixed thoroughly and then placed in a water bath heating pot (HH.S11–2, BOXUN, Shanghai, China) (set at 90 °C) without stirring and heated for 12 h. After heating, the solution was cooled to room temperature, the upper semi-transparent solution was decanted, and the lower layer mixture was centrifuged at 2000 rpm for 10 min. The copper nanowires were washed with a water-ethanol mixture (water/ethanol ratio of 2:8) and then washed twice with pure ethanol to remove excess impurities. Finally, the washed copper nanowires were dried in a vacuum oven (BCZ–76, BOXUN, Shanghai, China) at 70 °C for 6 h to obtain low-oxidation copper nanowires.

### 2.3. Preparation of Copper Conductive Ink and 3D Printing of Copper Circuits

To prepare homogeneous copper nanowires conductive ink, the dried copper nanowires were first added into anhydrous ethanol and left for 24 h, followed by three ultrasonic washing cycles with anhydrous ethanol for purification. Then, a certain amount of copper nanowires was placed into a centrifuge tube, using ethylene glycol, isopropanolamine, and ethanol in a ratio of 1:2:2 as the solvent. Ethylene glycol solution and isopropanolamine solution were first added with stirring and ultrasonication, followed by ethanol. The copper nanowires were uniformly mixed with the solvent by stirring and ultrasonic oscillation, and conductive inks with solid contents of 10%, 20%, 30%, and 40% were prepared, respectively. The copper nanowire conductive ink was loaded into a syringe, and direct ink writing technology was used to print patterns on the surface of a polyimide (PI) substrate. After drying in air, the printed patterns were sintered in a tubular furnace (OTF−1500X, HF−Kejing, Hefei, China). The furnace was evacuated to −0.1 MPa for 10 min, then filled with high-purity argon gas until the pressure was 0.01 MPa. This process was repeated three times, and then all the air in the tube was removed. The sintering temperatures were set at 300 °C, 400 °C, and 500 °C. The heating program was set to raise the temperature from 20 °C to the target temperature at a rate of 10 °C/min, maintained for 30 min, and the samples were taken out after being cooled with the furnace to room temperature. The sintering heating of the printed patterns caused the solvent to evaporate, thus obtaining high-quality copper nanowire conductive circuits.

### 2.4. Characterization

The microstructure of copper nanowires was characterized using transmission electron microscopy (TEM, JEOL-2100, JEOL, Tokyo, Japan), the accelerating voltage is 200 kV. The surface morphology of sintered printed patterns was analyzed using scanning electron microscopy (SEM, MERLIN Compact, Zeiss, Oberkochen, Germany); the accelerating voltage is 20 kV. An optical digital microscope (DSX 510, OLYMPUS, Shinjuku City, Tokyo, Japan) was used to observe the microstructure of the sintered printed patterns and to test and measure the surface morphology and wire diameter of the samples. The dried copper nanowires samples were analyzed by an X-ray diffractometer (XRD, DX-2700, Dandong Haoyuan Instrument Co., Ltd., Dandong, China). The molecular structure of the dried copper nanowire samples was characterized by Fourier transform infrared spectroscopy (Nicolet 380, Thermo Electron, Madison, WI, USA), to identify chemical bonds and functional groups. The thermal properties of the materials were studied by simultaneous thermal analyzer (STA449C, NETZSCH-Gerätebau GmbH, Selb, Germany) under a nitrogen atmosphere in the temperature range from room temperature to 700 °C, and the thermal stability was evaluated by monitoring the weight change of the samples in real time.

The resistance of the flexible copper nanowires circuits was measured using a benchtop multimeter, and the resistivity was calculated according to the formula:

(1) ρ=R×A/L

where ρ is resistivity, *R* represents the measured resistance, *L* represents the line length, and *A* represents the cross-sectional area of the printed wire. The length and width of the conductive pattern were measured using a micrometer.

Adhesion tests were used to evaluate the performance of the copper nanowire ink circuits. Standard tape (Scotchs 810, 3 M) was pressed tightly on the circuit surface and quickly removed. Multiple adhesion tests were performed to analyze the adhesion stability between the printed circuit layer and the substrate. The flexible copper nanowire circuits were subjected to repeated bending tests with a fixed bending radius (1.5 cm) using a curved mold in order to investigate their electrical performance under repeated mechanical deformation. The circuits were immersed in deionized water, removed, and dried at the specified time. The waterproof performance was evaluated by measuring the resistance change at regular intervals.

### 2.5. Fluid Mechanics Simulation

Rheological testing is a method to characterize the viscoelastic properties of materials by measuring their deformation and flow behavior under external force. A rheometer (Haake Mars40, Thermo Scientific, Karlsruhe, Germany) was used to measure the rheological properties of the conductive ink. The cone-plate system was used for the test [34], and viscosity was measured at room temperature in the shear rate range of 0.01–1000 s^−1^. The flow curves and apparent viscosity changes were obtained, and the shear-thinning or shear-thickening behavior of the non-Newtonian fluid was analyzed [35].

The Herschel–Bulkley model was used as the constitutive model. Since there was an initial yield stress, the Herschel–Bulkley model was expressed as a piecewise function. When shear stress *τ* was less than *τ*_0_, the shear rate (*γ̇*) was 0. The function is as follows:

(2) $$\left\{\begin{array}{c} \tau \geq \tau_{0},\;\tau =\tau_{0}+k\gamma^{n}\\ \tau \leq \tau_{0},\;\gamma =0 \end{array}\right.$$

Analysis of the 3D printing process was carried out. In the fluid mechanics simulation of conductive inks, COMSOL Multiphysics 6.1 software was used to analyze the effect of ink properties on printing performance. The simulation was based on a boundary condition of 1.4 atm at the nozzle inlet, and the fluid behavior of a conical nozzle with an inner diameter of 1500 μm was analyzed [36].

ANSYS Fluent R18.2 software was also used for viscoplastic fluid simulation. The nozzle and computational domain were first built using SpaceClaim. Then, structured meshing was applied, with hexahedral meshes in the nozzle and tetrahedral meshes outside. The dynamic mesh method was used, which allows the computational mesh to adapt to boundary or internal region motion or deformation in the fluid domain, thus accurately solving flow problems under changing geometry [37].

For the equation setup, since the printing materials had low flow velocity and high viscosity, the Volume of Fluid (VOF) model was selected. It is a CFD technique used to track the interface between two or more immiscible fluids (for example, ink and air) by solving for the volume fraction of each fluid in every computational cell:

(3) Re=ρuL/μ<<2000

where *ρ* is fluid density (kg/m^3^), *u* is characteristic velocity (m/s, extrusion speed in this model), *L* is characteristic length (m, nozzle diameter), and μ is dynamic viscosity (Pa·s).

The Mach number (Ma) can be used as a criterion for judging the validity of the incompressible fluid assumption. When:

(4) Ma=u/c<<0.3

where *u* is the characteristic flow velocity (m/s, taken as the extrusion velocity in the present model), and *c* is the speed of sound (m/s, approximately 343 m/s in air), the fluid can be considered incompressible. Since the current model satisfies the incompressibility condition, both the compression work and viscous dissipation terms can be neglected in the energy equation.

Under the incompressible flow assumption, this study adopts a laminar multiphase flow model utilizing the VOF method while accounting for the effects of both gravity and surface tension. The governing equations employed in the numerical simulations comprise:

1. Mass Conservation Equation (Continuity Equation) For incompressible fluids with constant density, the continuity equation reduces to:

(5) ∇⋅u=0

where *u* represents the velocity vector (m/s).

2. Momentum Conservation Equation (Navier–Stokes Equation) Under the laminar flow assumption, where turbulent effects are neglected, the momentum equation can be expressed as:

(6) $$\rho (\partial u/\partial t+u\cdot \nabla u)=-\nabla p+\mu \nabla^{2}u+\rho g+F_{st}$$

where *ρ* denotes the fluid density (kg/m^3^), *p* represents the pressure (Pa), μ indicates the dynamic viscosity (Pa·s), *g* is the gravitational acceleration vector (m/s^2^), and $F_{st}$ signifies the surface tension source term (N/m^3^), which is exclusively present at the gas–liquid interface.

## 3. Results and Discussion

### 3.1. Synthesis of Copper Nanowires

**Figure 2:**
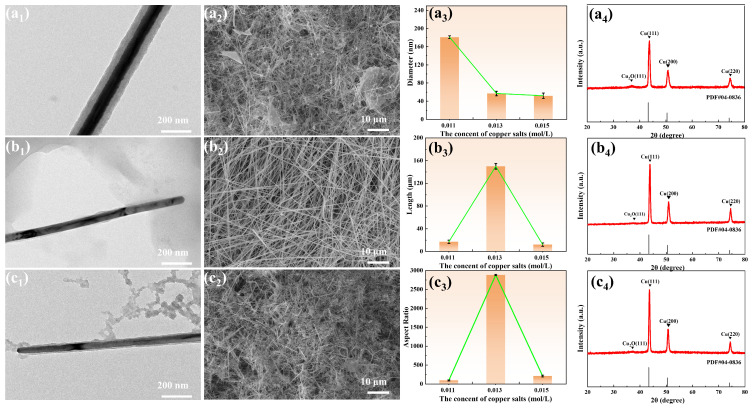
Effect of copper salt concentration on the morphology, size, and crystal structure of copper nanowires. (**a1**–**a4**) Sample 1 (copper chloride 0.011 mol/L), (**b1**–**b4**) Sample 6 (copper chloride 0.013 mol/L), (**c1**–**c4**) Sample 3 (copper chloride 0.015 mol/L).

The synthesis of copper nanowires is a chemical reaction in which the synthesis parameters significantly influence the nucleation and growth processes of the crystals. By precisely controlling the concentration of reactants, copper nanomaterials with high aspect ratios can be synthesized. The influence of copper salt concentration on the diameter, length, and aspect ratios of the copper nanowires is shown in Figure 2. As the concentration of copper chloride increases, the aspect ratios of the copper nanowires first increase and then decrease. At a copper salt concentration of 0.013 mol/L, the highest aspect ratio of 2884 was achieved, along with a relatively uniform overall morphology. To further investigate the crystal structure of the copper nanowires, X-ray diffraction (XRD) analysis was performed. Three characteristic peaks were observed at 2θ values of 43.24°, 50.44°, and 74.16°, corresponding to the (111), (200), and (220) crystal planes of face-centered cubic copper, respectively. No significant Cu_2_O diffraction peaks were detected. The results indicate that copper nanowires synthesized at different copper salt concentrations exhibit a lower initial degree of oxidation.

**Figure 3:**
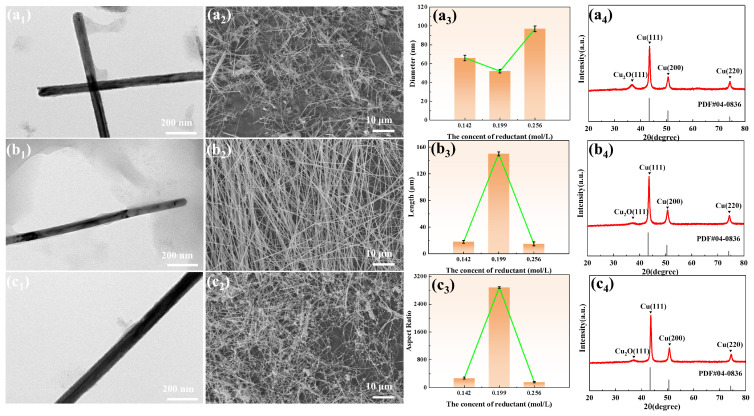
The influence of reducing agent concentration on the morphology, size, and crystal structure of copper nanowires. (**a1**–**a4**) Sample 4 (ascorbic acid 0.142 mol/L), (**b1**–**b4**) Sample 6 (ascorbic acid 0.199 mol/L), (**c1**–**c4**) Sample 5 (ascorbic acid 0.256 mol/L).

Figure 3 illustrates the effect of reducing agent concentration on the morphology, size, and crystal structure of the copper nanowires. When the concentration of ascorbic acid was 0.199 mol/L, the copper nanowires exhibited a well-defined morphology, uniform diameter, and the highest aspect ratio. Either too high or too low a concentration of ascorbic acid leads to a reduction in the aspect ratio and deterioration of the overall morphology. XRD results indicate that at lower reducing agent concentrations, the synthesized copper nanowires are oxidized to a greater extent. Increasing the reducing agent concentration effectively suppresses the appearance of Cu_2_O diffraction peaks, demonstrating enhanced oxidation resistance. This is attributed to the fact that at lower concentrations, insufficient reduction leads to some copper nanowires being prone to oxidation, while higher concentrations enhance the antioxidant property.

**Figure 4:**
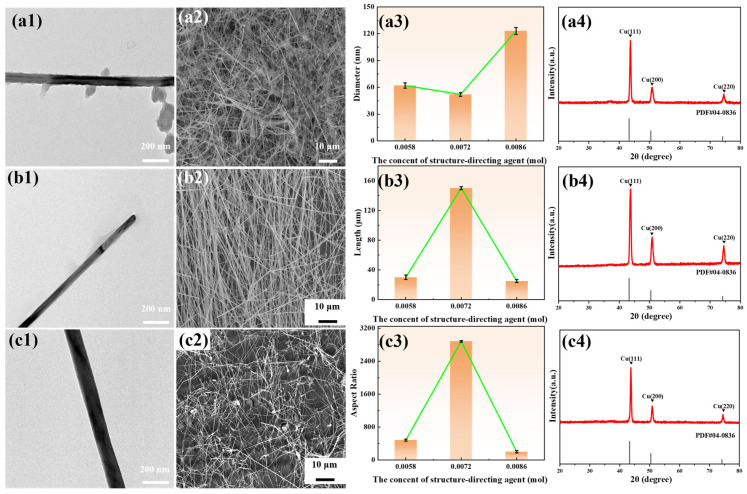
The influence of structure-directing agent concentration on the morphology, size, and crystal structure of copper nanowires. (**a1**–**a4**) Sample 2 (oleylamine 0.0058 mol/L), (**b1**–**b4**) Sample 6 (oleylamine 0.0072 mol/L), (**c1**–**c4**) Sample 7 (oleylamine 0.0086 mol/L).

Figure 4 demonstrates the influence of structure-directing agent concentration on the morphology, size, and crystal structure of the copper nanowires. When the oleylamine concentration was 0.0072 mol/L, the copper nanowires exhibited a relatively uniform morphology and achieved the highest aspect ratio of 2884. Both excessively high and low oleylamine concentrations result in reduced aspect ratios and non-uniform growth. Regardless of the concentration of the structure-directing agent, no significant oxidation peaks were observed in the XRD patterns. It is indicated that the synthesized copper nanowires possessed high oxidation resistance, and the concentration of the structure-directing agent had minimal influence on their oxidation behavior.

Based on the excellent morphology and high oxidation resistance, the concentrations of 0.013 mol/L copper chloride, 0.199 mol/L ascorbic acid, and 0.0072 mol/L oleylamine were identified as the optimal experimental parameters. Subsequent 3D printing and circuit tests were conducted using copper nanowires prepared under this specific formulation.

**Figure 5:**
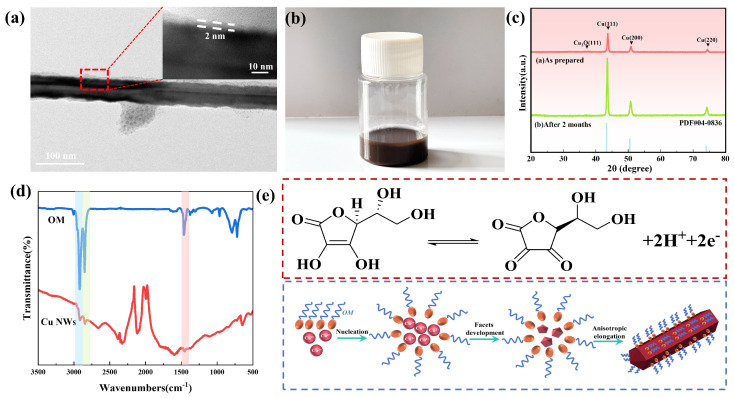
Stability, oxidation resistance, and formation mechanism of copper nanowires. (**a**) HRTEM image of copper nanowires, (**b**) conductive ink of copper nanowires, (**c**) X-ray diffraction (XRD) patterns of copper nanowires before and after 2 months of storage, (**d**) FT-IR spectrum of copper nanowires, (**e**) schematic illustration of the synthesis mechanism of copper nanowires.

The stability and oxidation resistance of copper nanowires are critical factors for their applications. As shown in Figure 5a, high-resolution SEM and TEM analyses revealed that the copper nanowires possessed a high aspect ratio [38,39]. Additionally, a surface coating of approximately 2 nm was observed, which effectively inhibited oxidation. In Figure 5b, the copper nanowires were dispersed in ethylene glycol to form a conductive ink, which showed no significant sedimentation after prolonged standing. Figure 5c demonstrates that the synthesized copper nanowires did not form copper oxide after storage under ambient conditions for two months. As shown in Figure 5d, FT-IR analysis of the copper nanowires displayed characteristic peaks at wavenumbers of 2920 cm^−1^, 2850 cm^−1^, and 1460 cm^−1^. These peaks fully match those of oleylamine molecules, confirming the adsorption of oleylamine on the surface of the copper nanowires, forming an anti-oxidation layer [40,41]. Figure 5e illustrates the proposed synthesis mechanism of the copper nanowires. Ascorbic acid reduces Cu^2+^ ions to Cu atoms by releasing electrons. The reduced copper atoms subsequently deposit and accumulate preferentially on the (111) crystal planes of the copper nanowires. Compared to the (111) planes, the (100) planes exhibit higher chemical reactivity [42], making them more susceptible to adsorption of the structure-directing agent. The amino group in oleylamine coordinates with copper atoms via coordination bonds, adsorbing onto the (100) planes and forming a dense coverage layer. As a result, the deposition of copper atoms on the (100) planes is suppressed, thereby inhibiting growth along this direction and controlling the morphology of the copper nanowires [43].

$$C_{6}H_{8}O_{6}⇌C_{6}H_{6}O_{6}+2H^{+}+2e^{-}$$

$$2e^{-}+Cu^{2+}⇌Cu^{0}$$

### 3.2. Preparation of Copper Conductive Ink and 3D Printing of Copper Circuits

The rheological properties of the conductive ink are crucial for achieving high-precision printing [44]. After 3D printing, the copper nanowire flexible circuits can be fabricated by combining with a sintering process [45]. Therefore, the investigation and optimization of key process parameters, such as printing settings and sintering temperature, are of great significance for producing flexible circuits with both high precision and excellent electrical conductivity.

**Figure 6:**
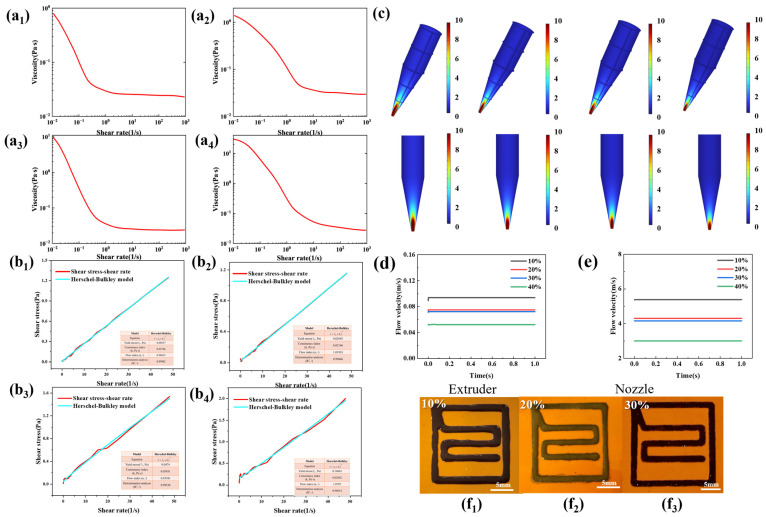
Fluid dynamics simulation and experiment of the extrusion process. Viscosity-shear rate of conductive inks with solid contents of (**a1**) 10%, (**a2**) 20%, (**a3**) 30%, and (**a4**) 40%, shear stress-shear rate curves and Herschel–Bulkley fitting curves of conductive inks with different solid contents, (**b1**) 10%, (**b2**) 20%, (**b3**) 30%, and (**b4**) 40%, (**c**) velocity distribution cloud diagrams of ink during extrusion for inks with different copper nanowires contents, (**d**) flow speed of inks in the hopper, (**e**) flow speed of inks at the nozzle exit, Printed patterns of copper nanowires inks with different solid contents, (**f1**) 10%, (**f2**) 20%, (**f3**) 30%.

As shown in Figure 6(a1–a4), the rheological properties of conductive inks with copper nanowire content of 10%, 20%, 30%, and 40% were tested. All inks exhibited shear-thinning behavior, where the viscosity decreased with increasing shear rate, thereby reducing the likelihood of nozzle clogging. Table 2 summarizes the key parameters of the Herschel-Bulkley model for these inks. The yield stress value (τ_0_) increased rapidly with higher CuNWs content.

Figure 6(b1–b4) shows that for inks with different copper nanowire contents, the correlation coefficients (R^2^) of the Herschel–Bulkley model are all relatively high, which indicates that the shear stress−strain curves are in good agreement with the Herschel–Bulkley fitting curve. Consequently, the parameters from this model were employed for subsequent 3D printing process simulations. Figure 6c demonstrates that the extrusion velocity of ink varied significantly with copper nanowire content. As the copper nanowires content increased, the flow rate of the ink gradually decreased. A quantitative analysis of the flow speed of inks with different copper nanowire contents is presented in Figure 6d,e. Both the velocity at the nozzle outlet and within the barrel decreased with higher CuNWs content. The ink with 10% copper nanowire content exhibited the highest flow velocity and the smallest ratio of nozzle outlet velocity to barrel velocity, suggesting the most stable flow behavior. Printed patterns from inks with different solid loadings are shown in Figure 6(f1–f3). When the solid content was low, the ink with low viscosity tended to spread during the printing process, resulting in unstable pattern definition and blurred edges. Increasing the solid content improved printing precision and morphological stability. However, at contents above 30%, the inks were prone to nozzle clogging during extrusion, leading to printing failure.

**Figure 7:**
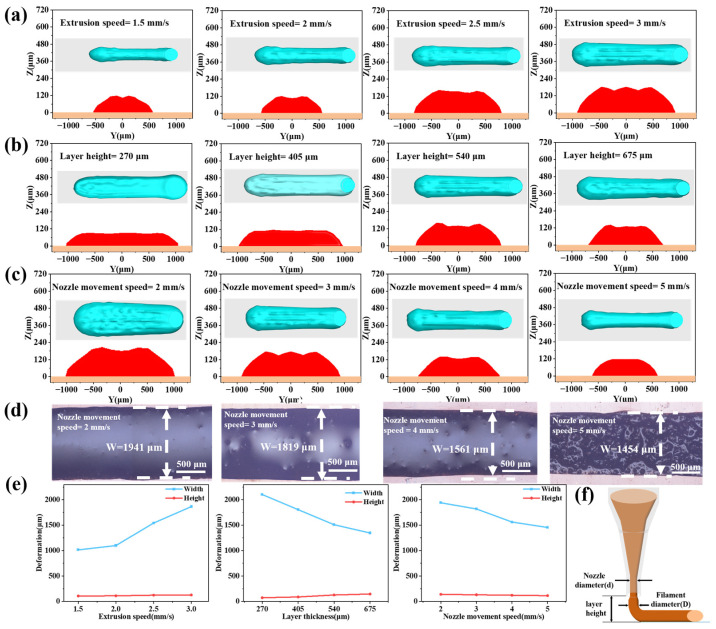
Fluid dynamics simulation of the 3D printing process. (**a**–**c**) The blue part is a top view of the simulated printed circuit, and the red part is a cross-sectional view., (**d**) filament morphology at different printhead moving speeds, (**e**) deformation under different extrusion speeds, different layer height, and different nozzle movement speeds, (**f**) printing layer height instructions.

Computational fluid dynamics simulations can predict the ink deposition process during 3D printing. Using the ANSYS platform, the influence of key process parameters (layer height, extrusion rate, and nozzle travel speed) on the morphology and structure of printed filaments was investigated. Figure 7a–c shows the shapes and cross-sections of printed filaments under different extrusion speeds, printing speeds, and layer heights. As shown in Figure 7a, a low extrusion speed resulted in a filament with a smaller cross-sectional width and an irregular shape due to insufficient ink flow. An increase in extrusion speed significantly increased both the cross-sectional width and height of the filament, and the contour became smoother. From Figure 7b, increasing the layer height led to a gradual decrease in the filament’s width but an increase in its height. Excessive layer height reduced the overall flatness of the printed filament. Figure 7c shows that increasing the nozzle movement speed reduced both the cross-sectional width and height of the filament. 3D printing experiments were conducted to further examine the effect of nozzle speed on printed line morphology; the results are shown in Figure 7d. The experimental results showed good agreement with the CFD simulations, indicating that the model can accurately guide the optimization of printing parameters.

Figure 7e quantifies the influence of different printing parameters on printed layer deformation. Increasing the extrusion speed increased the cross-sectional width of the extruded filament and slightly increased its height. Under a constant layer height, the filament, constrained between the nozzle and the substrate, spreads primarily in the width direction. Layer height had a notable impact on the cross-sectional shape: increasing the layer height decreased the width and increased the height of the filament. Increasing the nozzle movement speed significantly decreased the cross-sectional width of the extruded filament and slightly reduced its height.

**Figure 8:**
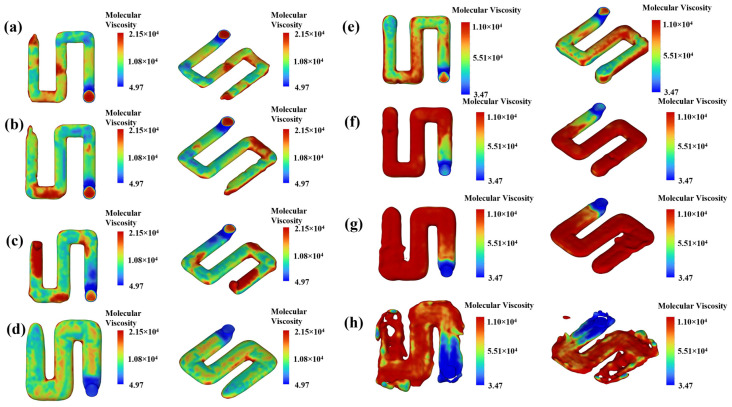
Viscosity variation of copper nanowires zigzag circuit under different printing layer heightes and printhead moving speeds. Layer height: (**a**) 405 μm, (**b**) 540 μm, (**c**) 675 μm, (**d**) 810 μm, printhead moving speed: (**e**) 2 mm/s, (**f**) 3 mm/s, (**g**) 4 mm/s, (**h**) 5 mm/s.

The results of printing a simulated zigzag circuit with the copper nanowires ink are shown in Figure 8. From Figure 8a–d, the ink viscosity was higher at the corners of the printed rectangular circuit. Increasing the printing layer height improves the geometric stability of the circuit’s contour. However, excessive layer height causes ink accumulation, blurring the pattern edges and reducing printing precision. As shown in Figure 8e–h, the speed of the ink increased significantly with higher nozzle speeds. Nevertheless, an excessively high nozzle speed causes pore formation on the circuit surface and disrupts the circuit shape. This is attributed to insufficient deposition of conductive ink per unit area, preventing the formation of a continuous and uniform structure [46], which severely compromises the circuit’s structural integrity and electrical performance. This study utilized CFD simulations to optimize the ink’s solid content and analyze the significant effects of process parameters—extrusion speed, nozzle speed, and layer height—on filament morphology, providing a basis for high-precision printing, ultimately yielding optimal parameters: nozzle movement speed of 3 mm/s, extrusion speed of 2.5 mm/s, and print layer height of 540 μm.

**Figure 9:**
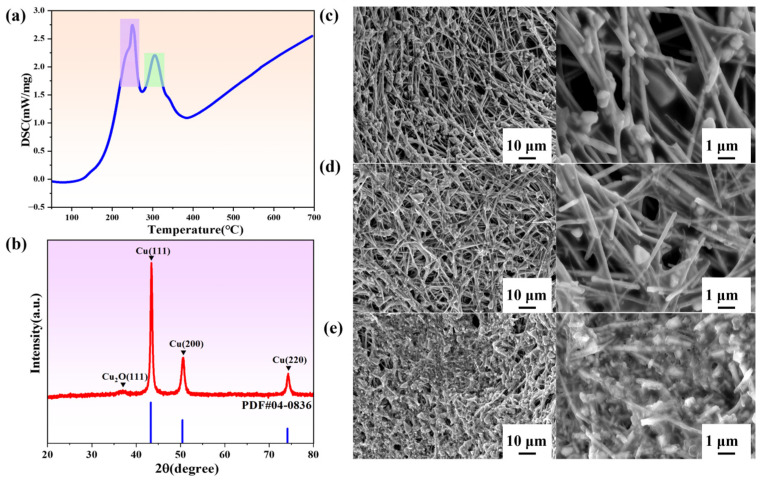
(**a**) DSC of copper nanowires, (**b**) XRD of copper nanowires flexible circuits after sintering, Influence of sintering temperature on the electrical properties of copper nanowires flexible circuits: (**c**) 300 °C, (**d**) 400 °C, (**e**) 500 °C.

The thermal behavior of the copper nanowires (CuNWs) was analyzed using simultaneous thermal analysis (STA). The results are shown in Figure 9a. The DSC curve of the CuNWs showed a distinct endothermic peak at approximately 250 °C, indicating the thermal decomposition of surface organics (confirming the presence of oleylamine) [47]. The endothermic peak near 300 °C is associated with the further decomposition of residual solvents. The results indicate that organic components in CuNWs can be effectively removed at temperatures between 300 and 400 °C, facilitating thermal sintering conditions. Figure 9b presents the XRD pattern of the flexible copper circuits after sintering. Only the characteristic diffraction peaks of copper are present, with no detectable oxide peaks, indicating that no oxidation reaction occurred during sintering in an argon atmosphere. This confirms that copper nanowires retain excellent electrical conductivity under these thermal sintering conditions.

The effect of sintering temperature on the microstructure of flexible CuNW circuits is shown in Figure 9c–e. As the sintering temperature increases, the point contacts between the copper nanowires gradually evolve into more obvious necking and interconnection structures. This enhances network continuity and reduces disconnections and interfacial contact resistance, thereby lowering the macroscopic resistance. At lower sintering temperatures, individual, unfused copper nanowires were observed, resulting in poor overall circuit connectivity. Increasing the sintering temperature improved the interconnection between most CuNWs. Upon further increasing the temperature to 500 °C, a complete and interconnected structure was formed, establishing a continuous conductive network. This is because the higher sintering temperature provides greater energy, accelerating the contact and fusion between adjacent CuNWs and thus promoting the formation of a denser interconnected structure [48].

### 3.3. Electrical Properties of Copper Nanowires-Based Flexible Circuits

**Figure 10:**
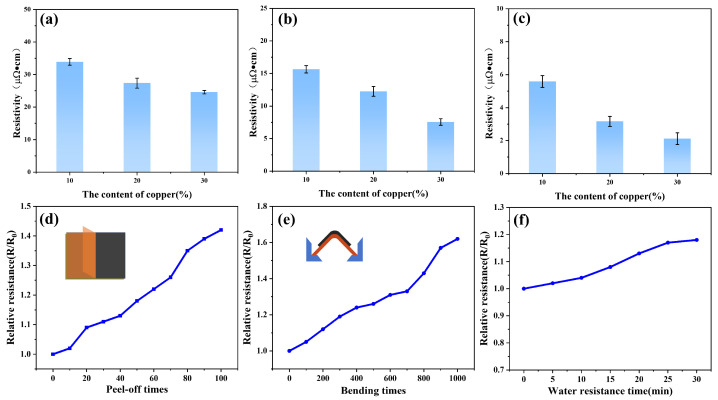
Resistivity of copper nanowires flexible circuits with different solid contents at different sintering temperatures: (**a**) 300 °C, (**b**) 400 °C, (**c**) 500 °C. Relative resistance (R/R_0_) of copper nanowires flexible circuits under (**d**) different peeling cycles, (**e**) different bending cycles, and (**f**) different water resistance durations.

Figure 10a–c presents the resistivity of flexible circuits fabricated with different solid contents of copper nanowires (CuNWs) sintered at various temperatures. At a fixed sintering temperature, the resistivity of the copper nanowires flexible circuits gradually decreases with increasing copper nanowire content. This is attributed to a reduction in inter-nanowire pores, which enhances the conductivity of the circuits [49]. The conductivity of circuits with different solid contents improves as the sintering temperature rises. This enhancement results from the progressively strengthened sintering and fusion between nanowires, leading to the formation of a more tightly conductive network [50]. When the sintering temperature reaches 500 °C, the resistivity of the copper nanowires flexible circuit drops to 2.1 μΩ·cm, which is only 1.2 times that of bulk copper (1.72 μΩ·cm). This indicates that the prepared flexible circuits possess excellent electrical conductivity.

Mechanical performance is a crucial factor in assessing the reliability of sintered metallic circuits, particularly for applications in flexible electronics. Copper nanowires flexible circuits must maintain stable electrical properties under various mechanical deformations, such as adhesion and bending. Figure 10d shows the results of peel tests performed on the copper nanowires flexible circuits. No significant residue of copper nanowires was observed on the testing tape during the process, demonstrating excellent adhesion between the copper nanowires flexible circuit and the substrate. To quantify the adhesion performance, the change in electrical resistance was evaluated by recording the relative resistance (R/R_0_, where R_0_ is the initial resistance before testing and R is the resistance during testing). Experiments reveal that the resistance of the copper nanowires flexible circuits increased gradually and then stabilized with an increasing number of peel cycles. After 100 peel cycles, the relative resistance (R/R_0_) increased only to 1.42, confirming its outstanding adhesion properties.

Figure 10e displays the mechanical performance of the copper nanowires flexible circuit during bending tests. Secure the printed flexible circuit to the cyclic bending tester and set the bending radius to a constant 1.5 cm for cyclic bending. When the number of bending cycles increased to 1000, the relative resistance increased merely to 1.62. This result indicates the circuit possesses high mechanical flexibility. This characteristic can be attributed to the dense structure formed between the circuit and the polyimide (PI) substrate, coupled with the strong adhesion.

Furthermore, waterproofing tests validated the circuit’s feasibility for practical applications. As shown in Figure 10f, the relative resistance increased by only a factor of 1.18 after the water immersion test, indicating excellent water resistance. This property is significant for protecting device functionality from impairment due to external environmental conditions in real-world applications. Through precise optimization of the copper nanowire synthesis process, nanowires with an aspect ratio of 2884 were achieved, exceeding the specifications commonly found in most studies on copper nanowires and certain commercial grades [51,52,53,54,55,56,57,58,59,60,61,62,63,64,65]. This implies that the percolation network of nanowires in transparent conductive films can be significantly enhanced, offering new possibilities for the application of high-performance copper nanowire-based materials.

In summary, conductive inks are pivotal materials for flexible printed electronics. Owing to their low cost and excellent conductivity, copper nanowires have emerged as an ideal candidate material for conductive inks. However, their propensity for oxidation and agglomeration severely challenges their printing performance. This study employed a liquid-phase reduction method, modulating the concentrations of ascorbic acid and oleylamine to synthesize copper nanowires with high aspect ratios and high oxidation resistance. A solvent system comprising isopropanolamine, ethylene glycol, and ethanol was introduced to formulate a stable and oxidation-resistant copper nanowire ink. The influence of ink composition and printing parameters on printing precision was investigated through a combination of hydrodynamic simulation and experimental validation, enabling high-precision printing. Finally, flexible circuits were obtained through thermal sintering, and their mechanical properties were evaluated. This study successfully fabricated flexible copper nanowire circuits with high precision and high conductivity, which holds significant importance for advancing the development of wearable electronic devices.

## 4. Conclusions

In this study, a copper nanowires conductive ink with high precision, high conductivity, and oxidation resistance was synthesized. Copper nanowires materials with a high aspect ratio were prepared by a liquid-phase reduction method, achieving an aspect ratio of 2884, with favorable morphology and excellent antioxidant properties. Using ethylene glycol, isopropanolamine, and ethanol as solvents, a conductive ink with excellent viscosity and stability was formulated. Precise regulation of the copper nanowires ink during 3D printing was achieved by combining hydrodynamic simulation with experiments. The effect of sintering temperature on the prepared flexible circuits was investigated. When the sintering temperature was 500 °C, the resistivity of the copper nanowires flexible circuit was only 2.1 μΩ·cm, which is merely 1.2 times that of bulk copper. Moreover, after 100 peeling cycles and 1000 bending cycles, the relative resistance (R/R_0_) of the circuit remained as low as 1.42 and 1.62, respectively. The relative resistance increased by only 1.18 times after 30 min of water exposure. These results demonstrate that the 3D-printed flexible circuits based on copper nanowires exhibit an overall highly stable state, coupled with high conductivity, strong adhesion, and exceptional mechanical flexibility. These findings indicate that this study has successfully addressed the key technical challenges of easy agglomeration and oxidation of copper nanowires. The work shows outstanding potential for achieving high-precision and well-controllable manufacturing of copper nanowires-based flexible circuits, holding significant practical value for advancing the innovation and development of wearable electronic devices.

## Figures and Tables

**Table 1:** Synthesis of copper nanowires under different reaction parameters.

Sample	Cupric Chloride (mol/L)	Ascorbic Acid (mol/L)	Oleylamine (mol)
1	0.011	0.199	0.0058
2	0.013	0.199	0.0058
3	0.015	0.199	0.0058
4	0.013	0.142	0.0058
5	0.013	0.256	0.0058
6	0.013	0.199	0.0072
7	0.013	0.199	0.0086

**Table 2:** Herschel–Bulkley model parameters of inks with different copper nanowires contents.

Copper Nanowires Content	Yield Stress (τ_0_, Pa)	Consistency Coefficient (k, Pa·sⁿ)	Flow Behavior Index (n, Dimensionless)
10%	0.00537	0.02746	0.98635
20%	0.02645	0.02196	1.01918
30%	0.0474	0.03931	0.93381
40%	0.16863	0.02842	1.0707

## Data Availability

The original contributions presented in this study are included in the article. Further inquiries can be directed to the corresponding authors.
